# Development and Formative Evaluation of a Low-Fidelity Equine Castration Model for Veterinary Education

**DOI:** 10.3389/fvets.2021.689243

**Published:** 2021-09-14

**Authors:** M. Katie Sheats, Megan J. Burke, James B. Robertson, Katherine E. Fiebrandt, Callie A. Fogle

**Affiliations:** North Carolina State University College of Veterinary Medicine, Raleigh, NC, United States

**Keywords:** competency, veterinary education, veterinary simulators, surgical simulation and training, model development and evaluation, equine castration model, horse castration model

## Abstract

Entrustable Professional Activities (EPAs) are units of activity that early-stage professionals perform in the workplace that necessitate simultaneous integration of multiple competencies. EPA #6 requires students to perform a common surgical procedure on a stable patient, including pre-operative and post-operative management. Castration is one of the most common surgeries performed by equine primary care practitioners and is considered an “entry-level competency” for veterinary graduates entering equine private practice, however, to our knowledge there are no equine castration models available for veterinary student education. Therefore, we developed an inexpensive, low-fidelity model of equine field castration and evaluated it using a mixed-methods approach. Two different groups of students, with or without model experience, completed surveys before and after live horse castration. Students who used the model also completed model specific surveys. Videos of the students completing the model were evaluated by at least two different equine veterinary faculty using a 15-point rubric, and inter-rater reliability of the rubric was determined. After completing the model, students reflected on strengths and weaknesses of their performance. From our student survey results, we determined that student attitudes toward the model were mostly positive. Interestingly, there were several student attitudes toward the model that became significantly more favorable after live horse castration. Prior to live horse castration, there was no significant difference in confidence in model vs. no-model groups. Following live horse castration, students who used the model had higher confidence in procedure preparation and hand-ties than students who did not use the model, but they had lower scores for confidence during patient recovery. When reflecting on model castration, students most commonly cited preparation and surgical description as strengths, and ligature placement and hand-ties as weaknesses. Experts provided several suggestions to improve the model, including incorporation of emasculators and the need for better model stabilization. Our findings suggest that both students and veterinary educators feel that this low-fidelity model has educational value. Rubric performance metrics were favorable, but additional steps are needed to improve grading consistency among educators. Future research will determine whether student performance on the model is predictive of competence score during live-horse castration.

## Introduction

Castration is one of the most common surgeries performed by equine primary care practitioners and is considered an “entry-level competency” for veterinary graduates entering equine private practice (1, 2). Castration surgical errors and post-operative complications are well-documented and range from excess swelling and incomplete castration to life-threatening evisceration, penile trauma, and/or hemorrhage (3–6). These complications are among the most common causes of malpractice claims against equine practitioners in North America (6). Given the routine nature of this procedure, its potential impact on animal health, and the potential for malpractice claims against the veterinary practitioner, it is incumbent upon veterinary educators to ensure “day 1-ready” veterinary graduates can competently perform routine equine field castration. While the curriculum at many veterinary schools does allow students to observe and even practice live-horse castration, the number of times students perform this surgery can vary and the student stress-level can present a barrier to learning. Therefore, there is a need for alternative educational approaches for teaching and assessing this Entrustable Professional Activity (7).

Small animal surgical simulators, such as those described for feline and canine ovariohysterectomy, allow students multiple opportunities to practice routine veterinary surgeries in a low-stakes environment without the use of animals (8–10). Recent reports on newly developed canine and bovine castration models show positive survey feedback from both veterinary students and clinicians, with both groups reporting that models were useful for surgical skills training (11, 12). Hunt et al. reported that student performance scores on a canine pre-scrotal closed castration model were strongly correlated with their live castration performance (11). Similarly, Anderson et al. reported that veterinary students trained on a bovine castration model had higher performance scores on live-bull calf castration than students who received only a traditional lecture (12). Clearly, veterinary models can be used to support student training when case numbers, or animal resources, are limited. Models also offer students a safe and inexpensive way to practice a multi-competency activity at the performance level of Miller's pyramid (13).

Given the wealth of evidence for the potential educational, ethical, practical, and financial advantages of models/simulators for veterinary student surgical training (9, 11, 12, 14–16), and the lack of an available equine castration model, we sought to develop a low-fidelity model of closed equine castration as performed on a “cast” or recumbent horse. The specific goals for this project were to create an affordable simulator and associated formative assessment that allowed students a chance to: (1) practice a cognitive, step-wise approach to equine field castration, (2) recognize their own strengths and weaknesses when performing a clinical procedure, and (3) receive timely and specific expert feedback on this skill. This manuscript describes the construction of the low-fidelity model, the grading rubric and inter-rater rubric performance, and student and expert survey feedback on the model. While most of our survey items were quantitative, we also sought open-ended feedback from students and veterinarians in order to identify opportunities for model improvement.

## Materials and Methods

**Figure 1:**
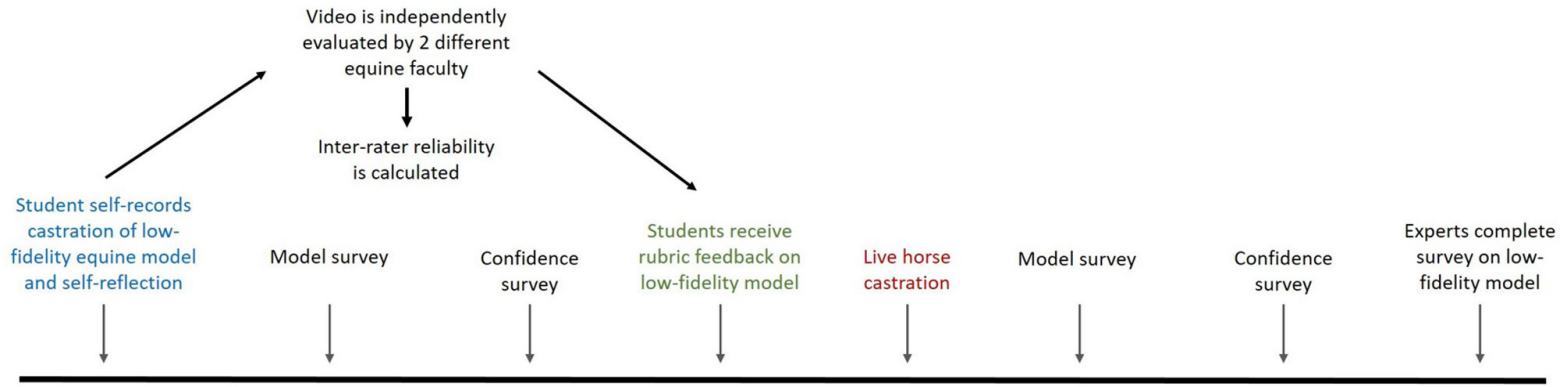
Study design. As part of a third year course on equine veterinary field skills, students recorded themselves performing a “closed” castration on a low-fidelity equine model. The students also recorded two strengths and weaknesses as part of a self-reflection assignment. The student videos were independently scored by 2 different equine faculty using a proposed rubric. Also as part of the course, students were asked to complete 2 surveys before, and after, participation in field castration of a live horse. Following completion of the castration model activity, students received their rubric score. Following student data collection, veterinary experts were asked to evaluate the model via survey.

This study was approved by the North Carolina State University Institutional Review Board (IRB #9541). An overview of the study approach is provided in Figure 1.

### Model Development

**Figure 2:**
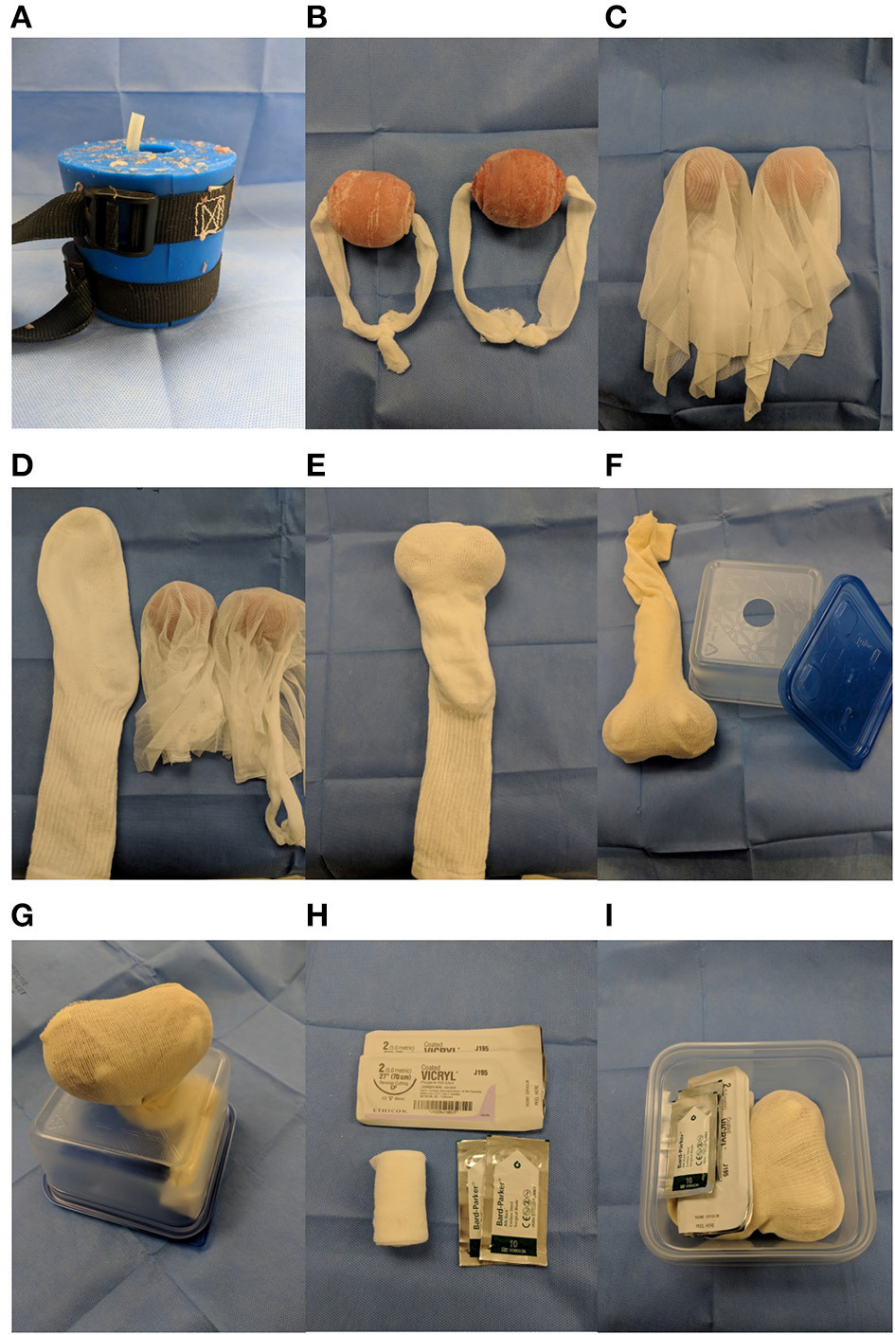
Overview of making and assembling components of low-fidelity equine castration model. Mold used to pour silicone testicles with rubber tubing to create a hole in the center **(A)**. Silicone testicles threaded with 18 inch length of 2 inch conforming stretch gauze bandage material to serve as the spermatic cord **(B)**. Testicles draped with stretchable mesh fabric (cut in 15 inch diameter circles) to represent the vaginal tunic **(C)**. Large men's tube sock containing side-by-side testicles **(D,E)**. Upside-down 42 ounce re-usable food container with open-end of tube sock inserted through 1.25 inch hole **(F,G)**. Suture and blades included as part of castration model kit **(H)**. All components assembled for distribution to students **(I)**.

The model for recumbent, closed equine castration was developed by veterinarians involved in teaching the skill of castration at NCSU, together with simulation laboratory and teaching technicians (Figure 2). Priorities for the model were to select components that would enable repeated practice with preparation, sterility, skin incision, testicle exteriorization, and ligature placement. A 42-ounce re-usable food container was used, upside down, as the base for the model (Figure 2F). An oblong clay model of an average yearling-sized testicle was used to create a mold, which was then used to create re-usable silicone testicles (Figure 2B). At the time of pouring, a tube was placed down the center of the silicone to allow for a 18 inch length of replaceable two inch conforming stretch gauze bandage material to be threaded through, to serve as the spermatic cord (Figure 2A). Each silicone testicle and associated gauze cord was then encased in a stretchable mesh fabric (cut in 15 inch diameter circles), similar to sheer tights or stockings, to represent the vaginal tunic encasing the testicle and contents of the spermatic cord (Figures 2C,D). Two mesh covered testicle/cord constructs were then placed into a men's large tube sock to serve as the scrotum (Figure 2E). The open end of the tube sock was threaded through a 1.25-inch hole cut in the center of the food container (Figure 2G). Care was taken to make sure that the cord gauze, mesh tunic, and sock scrotum were of sufficient length within the food container, so that the ends of all three components remained within the container during the castration procedure. Kits were also supplied with 2 suture packs of #2 Vicryl on a reverse cutting needle and two #10 scalpel blades (Figure 2H). All model components fit into the plastic food container for storage and distribution to students (Figure 2I).

### Survey of Students Without Castration Model Experience

Prior to development of the equine castration model, we collected voluntary, anonymous surveys from a convenience sample of 14 equine-interested DVM students (years 1–3) with no castration model experience, in order to better understand student confidence regarding castration of live horses in the field. These students completed pre- and post-live horse castration surveys (Boxes 1, 2) as part of an extracurricular wet lab in which each student participated in anesthetizing and castrating a live horse.

Box 1 **Student confidence survey prior to live horse castration.**

Please indicate how strongly you agree or disagree with the following statements:

1 = *strongly agree*, 2 = *disagree*, 3 = *neutral*, 4 = *agree*, 5 = *strongly agree*

1. I am technically competent to perform castration today.

2. I am mentally confident to perform castration today.

3. I am knowledgeable of the anatomical structures involved in equine castration.

4. I am knowledgeable of the important history questions and post-op complications relevant to equine castration.

5. Please report the number of individual testicles you have previously removed from a horse.

6. Please report the total number of equine general anesthesia events you have been involved in (i.e., observed, participated, conducted, etc.).

Box 2 **Student confidence survey post-castration.**

Please indicate how strongly you agree or disagree with the following statements:

1 = *strongly agree*, 2 = *disagree*, 3 = *neutral*, 4 = *agree*, 5 = *strongly agree*, 6 = *not applicable*

1. I feel my preparation for today's procedure was adequate.

2. Overall, I felt confident performing general anesthesia today.

3. Overall, I felt confident performing equine castration today.

4. I had anxiety while performing general anesthesia today.

5. I had anxiety while performing castration today.

6. I would have known what to do today if my patient started waking up from anesthesia during surgery.

7. I felt confident while recovering my patient.

8. I was knowledgeable today on concepts of sterile technique.

9. I was confident today in my knowledge of reproductive anatomy.

10. I was confident today in my hand-tie skills.

11. I was confident today in my ligature placement and security.

12. I would have known what to do today if my patient had bleeding from the surgical area.

13. I am confident that I know how to give clients accurate instructions regarding discharge care, monitoring and potential complications for equine castration.

14. Please report the number of individual testicles you have removed from a horse including today's procedure.

15. Please report the total number of equine general anesthesia events you have been involved in (i.e., observed, participated, conducted, etc.).

### The Low-Fidelity Castration Simulator Assignment

**Figure 3:**
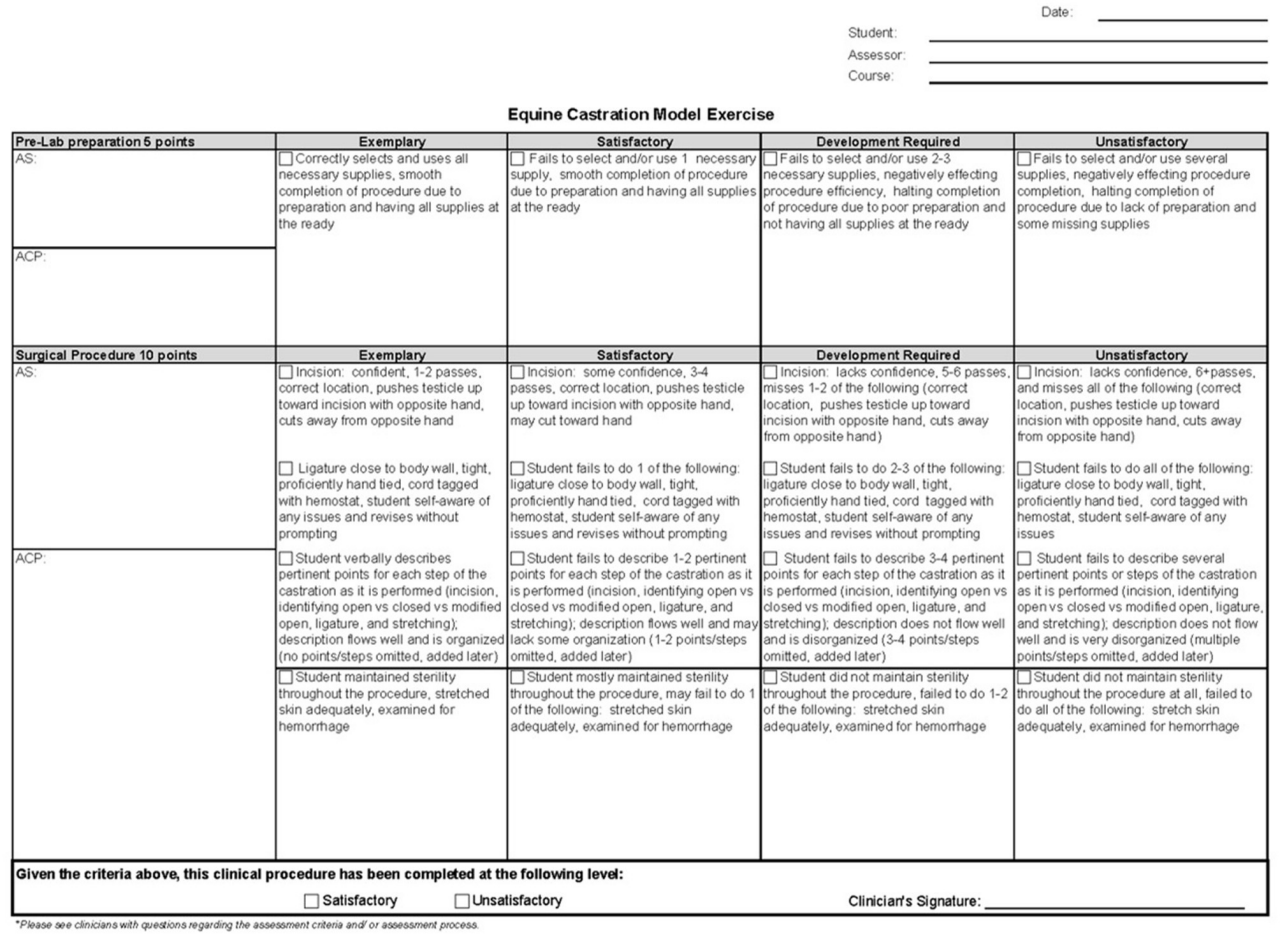
Low-fidelity castration model rubric. Exemplary = 3 points, Satisfactory = 2 point, Development required = 1 point, Unsatisfactory = 0 points. AS, areas of strength; ACR, areas for concentrated practice.

The castration simulator activity, including the model (Figure 2), associated activity instructions (Supplementary Document 1), and grading rubric (Figure 3), were developed and introduced to a third year DVM equine elective course in 2017 as a formative, low-stakes assessment. Students received a lecture and assigned reading on equine surgical castration. Specific for the assignment, students were instructed to review a video of a veterinary surgeon (CF) completing and narrating the castration model (Supplementary Video 1). They were then instructed to record themselves castrating the model while explaining key steps of equine castration. Finally, students were instructed to separately submit a reflection video where they identified 2 strengths and 2 weaknesses of their model castration. Students had access to the grading rubric as part of the course materials. After all rubric grades were complete, one investigator (MKS) watched the self-reflection videos and recorded the number of students identifying strengths or weakness in 8 different categories. Category descriptions were selected to encompass general themes of student responses including: preparation (i.e., having all of the materials ready and organized), incision (i.e., number of passes with the scalpel, length of incision, etc.), hand ties (i.e., proficient, not proficient), ligature (i.e., location on “cord,” too loose, etc.), surgical description (i.e., complete vs. incomplete, sequential, etc.), addressed complications (i.e., student related aspects of procedure to prevention of complications), sterile technique (i.e., did or didn't maintain sterility), and speed of procedure, which students could have reflected on as being appropriate, too fast or too slow.

In 2017 and 2018 the student model castration videos were randomly assigned to, and evaluated by, 2 of 3 equine veterinary faculty educators (MB, CF, and MS) using a 15-point rubric (Figure 3), so that each student received 2 independent scores. Because it was a video with audio, reviewers were not blinded. The rubric assigned 5 points to pre-lab preparation and 10 points to surgical procedure for a maximum possible score of 15. Pre-lab preparation addressed organization of required supplies, while surgical procedure addressed incision, ligature, verbal description, and sterility. Within the subcategories were brief descriptions that assisted instructors in assigning student performance as unsatisfactory (0 points), development required (1 point), satisfactory (2 points), and exemplary (3 points). Instructors were also asked to provide comment on Areas of Strength (AS) and Areas for Concentrated Practice (ACP). Instructors were blinded to other reviewer scores until all reviews were complete.

### Survey of Students With Castration Model Experience

As part of our process to develop and refine the equine castration simulator, we conducted voluntary surveys of students enrolled in the elective equine field skills course in 2018 and 2019. All students involved in this study were over the age of 21 and had completed at least 3 years of undergraduate education. After completing the model, but before castrating a live horse (as part of the course), students were surveyed on the model (Box 3) and their confidence (Box 1) to perform live horse castration. After live horse castration, students were given a similar survey on the model (Box 4), and on their confidence while performing live horse castration (Box 2). Grounded theory emergent coding was used by one investigator (KF) to identify themes in student open-ended comments about the model. A second investigator (MS) conducted a validity check on codes. Minor changes to codes were resolved collaboratively.

Box 3 **Model survey completed by students prior to live horse castration.**

Please select all of the following that describe your previous experience with equine field castration:

Instructional videos

Instructional text/articles

Observed live horse castration

Assisted with live horse castration

Performed live horse castration (in part or in whole) in private practice

Performed live horse castration (in part or in whole) with supervision of university faculty

Castration simulation model

Other

Please evaluate your experience using the following scale:

1 = *strongly agree*, 2 = *disagree*, 3 = *neutral*, 4 = *agree*, 5 = *strongly agree*

Performing castration on the model

1…. increased my knowledge of the surgical procedure.

2…. increased my knowledge of the key anatomical structures.

3…. was helpful for learning the surgical technique of routine equine castration.

4…. increased my confidence to perform live horse castration.

5. Instructions for the equine castration model were straightforward.

6. The equine castration model was easy to use.

7. Simulation models should look and feel realistic in order to teach students technical skills.

8. The equine castration model helped me identify my areas of strength and weakness for this skill.

9. I feel confident to perform routine equine field castration under experienced supervision.

Comments: Please write any feedback or suggestions you have about the equine castration model.

Box 4 **Model survey completed by students after live horse castration.**

Please select all of the following that describe your previous experience with equine field castration:

Instructional videos

Instructional text/articles

Observed live horse castration

Assisted with live horse castration

Performed live horse castration (in part or in whole) in private practice

Performed live horse castration (in part or in whole) with supervision of university faculty

Castration simulation model

Other

Please evaluate your experience using the following scale:

1 = *strongly agree*, 2 = *disagree*, 3 = *neutral*, 4 = *agree*, 5 = *strongly agree*

Performing castration on the model

1…. increased my knowledge of the surgical procedure.

2…. increased my knowledge of the key anatomical structures.

3…. was helpful for learning the surgical technique of routine equine castration.

4…. increased my confidence to perform live horse castration.

5. Simulation models should look and feel realistic in order to teach students technical skills.

6. The equine castration model helped me identify my areas of strength and weakness for this skill.

7. Performing castration on the model was easier than performing castration on the live horse.

8. I feel confident to perform routine equine field castration under experienced supervision.

Comments: Now that you have performed live horse castration, please write any feedback or suggestions you have about the equine castration model.

As part of a secondary analysis, student survey responses were used to divide survey data into Tiers I–III to indicate students' previous experience with equine castration (see Table 1).

**Table 1:** Student groups based on previous castration experience.

**Tier**	**Description**
Tier I	Student has not previously removed any testicles from a live horse.
Tier II	Student has previously removed 1-2 testicles from a live horse.
Tier III	Student has previously removed 3 or more testicles from a live horse.

### Veterinarians' Evaluation of the Model

Veterinary educators ranging from senior faculty to large animal surgery residents (*n* = 10) were recruited to provide feedback on the model and rubric. Veterinarians were given the same instructions the students received, asked to complete the castration model as a student participant would, and then asked to complete an anonymous 5 point Likert-scale survey (1 = *strongly disagree* and 5 = *strongly agree*), as well as provide comment (see Box 5).

Box 5 **Model survey completed by veterinary experts.**

Please evaluate your experience using the following scale:

1 = *strongly agree*, 2 = *disagree*, 3 = *neutral*, 4 = *agree*, 5 = *strongly agree*

1. The overall size of the model was appropriate for the skill.

2. The model was straightforward and easy to use.

3. The relevant anatomical structures were adequately represented.

4. The model was suitable to teach the basic steps required to perform the technical skill of equine castration to a beginner student.

5. I feel that this model will be helpful for students to practice before performing castration on a live horse.

6. I feel that the rubric and model together assess the students on the most important parts of routine equine field castration.

7. I have concerns that this model could teach students poor technique.

8. I feel that simulation models need to be very realistic in order to teach students technical skills.

9. I feel that I could easily use the rubric and student videos to assess the skills and knowledge of students using this castration model.

Please indicate your level of veterinary training.

Intern

Resident

Academic veterinarian

Private practice veterinarian

Comments: Please provide any additional comments.

### Statistical Analysis

Survey data were analyzed using descriptive statistics such as median, mean, and standard deviation. For comparison between no model vs. model experience, and pre- vs. post-live horse castration, survey results were tested for normality (D'Agostino & Pearson test) and analyzed using either unpaired or paired two-sided *t*-test (parametric data) or Mann-Whitney *U*-test (non-parametric data), respectively. For Tier I-III comparison, survey results were determined to be non-normally distributed (D'Agostino & Pearson test) and analyzed by Kruskal-Wallis test. Significance was set at *P* < 0.05 and analyses were performed using Graphpad Prism 9.0.

Rubric data were grouped by evaluator (MB, CF, or MS) and analyzed using descriptive statistics such as median, mean, and standard deviation. Data were tested for normality (D'Agostino & Pearson test) and analyzed by Kruskal-Wallis test, with *P* < 0.05 set as significant. Analyses were performed with Graphpad Prism 9.0. As a measure of inter-rater reliability, intraclass correlation coefficient with a two-way model (random subjects, random reviewers) was calculated using the irr package in R version 4.0 (17).

## Results

### Model Development and Cost

The cost for materials to make up one low fidelity castration model was $32 USD. Other than the silicone and mold used to make the reusable testicles, all components of the model are easy to obtain commercially. During the emasculation step of the castration procedure, all components of the model with the exception of the plastic food container, and the silicone testicles are transected, necessitating replacement prior to reuse of the model. Replacement of these non-reusable parts is approximately $25 USD, with the #2 Vicryl representing the largest cost at about $10/package. The plastic food container and silicone testicles do not break down or lose functionality with repeated use, and can be used an unlimited number of times without requiring replacement.

### Quantitative and Qualitative Model Feedback From Students

Of the 32 students enrolled in the equine field skills elective in 2018 and 2019, 32 completed the pre-live horse castration surveys and 31 completed the post-live horse castration surveys. Prior to live horse castration as a part of the course, there with 13 students with no prior castration experience (Tier I), 8 students who had castrated 1–2 testicles (Tier II), and 11 students who had castrated 3 or more testicles (Tier III).

**Figure 4:**
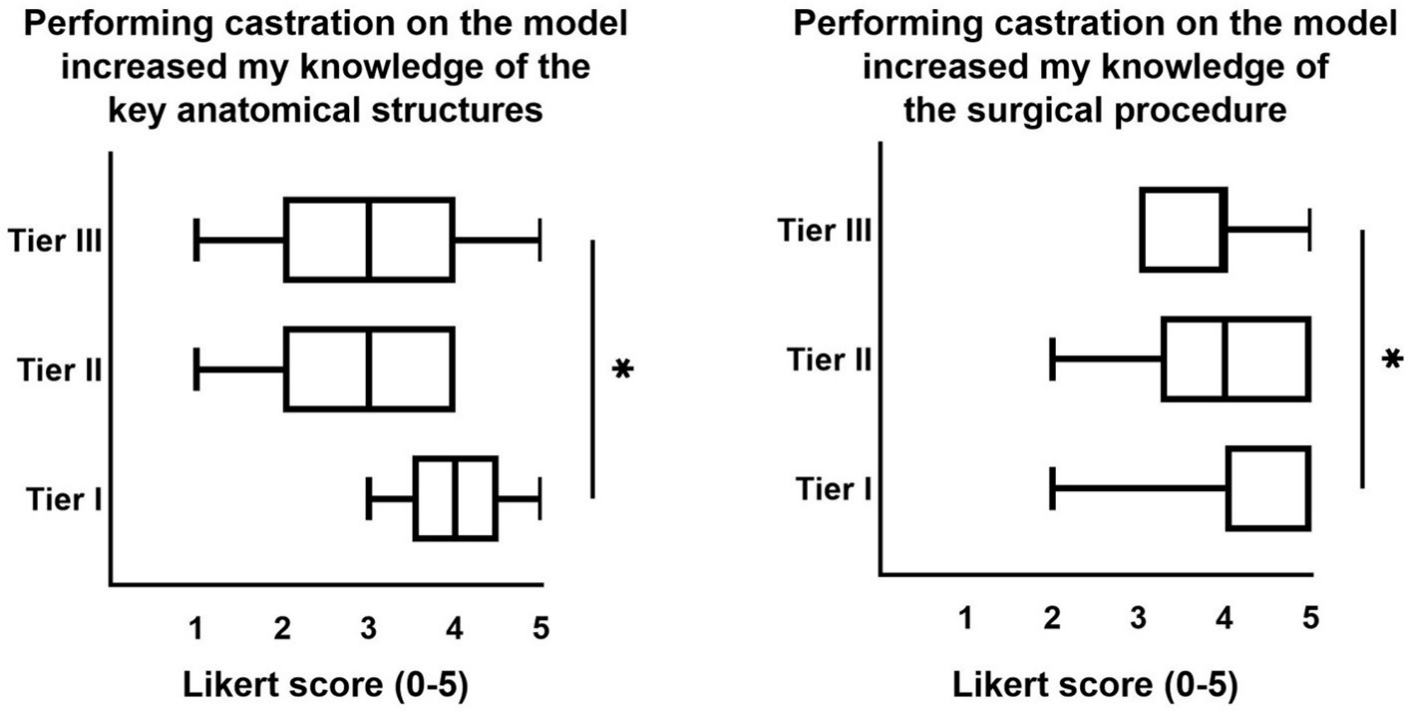
Significant differences in Likert score model ratings in students with different levels of castration experience, prior to live horse castration. Heading is the text taken from the survey prompt. Data are presented as median and min-max. Statistical analysis performed with Kruskal-Wallis test with Dunn's multiple comparisons *post-hoc* test. *p < 0.05 indicates significant difference between Tier I (no prior castration experience) and Tier III (3 or more testicles previously castrated).

**Figure 5:**
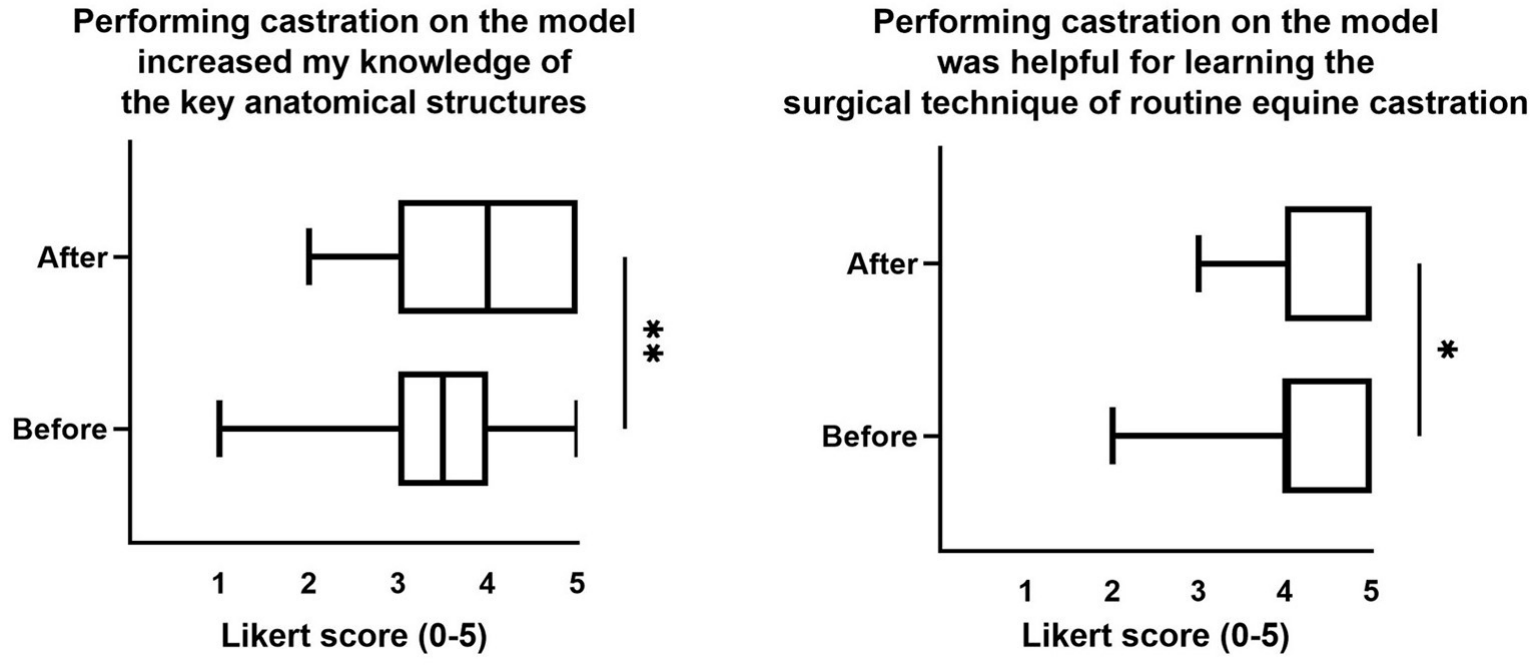
Significant differences in Likert score model ratings from students before vs. after live horse castration. Heading is the text taken from the survey prompt. Data are presented as median and min-max. Statistical analysis performed with Kruskal-Wallis test with Dunn's multiple comparisons *post-hoc* test. *p < 0.05 and **p < 0.005.

Overall student ratings of the model both before (Table 2) and after (Table 3) live horse castration were positive. Prior to live horse castration, students with the least experience (Tier I) had more favorable attitudes toward the model for increasing knowledge of “the surgical procedure” and “key anatomical structures,” compared to students with the most experience (Tier III) (Figure 4). This difference was not observed in the survey following live horse castration (data not shown). After live horse castration, total student responses were significantly more favorable toward the model for “increasing knowledge of key anatomical structures” and being “helpful for learning the surgical technique of routine equine castration” (Figure 5). Following live horse castration, there were no significant differences in model survey responses among students with different levels of castration experience (data not shown).

**Table 2:** Student assessment of the model prior to live horse castration.

**Survey**	**Strongly**	**Disagree**	**Neutral**	**Agree**	**Strongly**	**Mean**	***SD***	**Median**	***n***
**item**	**disagree *n* (%)**	***n* (%)**	***n* (%)**	***n* (%)**	**agree *n* (%)**				
**Performing castration on the model**									
1…. increased my knowledge of the surgical procedure.		2 (6.1%)	6 (18.2%)	10 (31.3%)	14 (43.8%)	4.125	1.096	4	32
2…. increased my knowledge of the key anatomical structures.	2 (6.3%)	5 (15.6%)	9 (28.1%)	12 (37.5%)	4 (12.5%)	3.344	0.8958	3.5	32
3…. was helpful for learning the surgical technique of routine equine castration.		2 (6.3%)	4 (12.5%)	12 (37.5%)	14 (43.8%)	4.188	1.051	4	32
4…. increased my confidence to perform live horse castration.	2 (6.3%)		8 (25%)	13 (40.6%)	9 (28.1%)	3.844	0.6927	4	32
5. Instructions for the equine castration model were straightforward.		1 (3.1%)	2 (6.3%)	19 (59.4%)	10 (31.3%)	4.188	0.9873	4	32
6. The equine castration model was easy to use.	1 (3.1%)	2 (6.3%)	6 (18.8%)	15 (46.9%)	8 (25%)	3.844	1.04	4	32
7. Simulation models should look and feel realistic in order to teach students technical skills.		2 (6.3%)	9 (28.1%)	4 (12.5%)	17 (53.1%)	4.125	0.9158	5	32
8. The equine castration model helped me identify my areas of strength and weakness for this skill.	1 (3.1%)		4 (12.5%)	12 (37.5%)	15 (46.9%)	4.25	0.5599	4	32
9. I feel confident to perform routine equine field castration under experienced supervision.			1 (3.1%)	11 (34.4%)	20 (62.5%)	4.594	1.096	5	32

**Table 3:** Students' assessments of the model after to live horse castration.

**Survey item**	**Strongly disagree *n* (%)**	**Disagree *n* (%)**	**Neutral *n* (%)**	**Agree *n* (%)**	**Strongly agree *n* (%)**	**Mean**	***SD***	**Median**	***n***
**Performing castration on the model**									
1…. increased my knowledge of the surgical procedure.			6 (18.2%)	9 (31.3%)	16 (43.8%)	4.323	0.7911	5	31
2…. increased my knowledge of the key anatomical structures.		3 (9.7%)	9 (29%)	9 (29%)	10 (32.3%)	3.839	1.003	4	31
3…. was helpful for learning the surgical technique of routine equine castration.			3 (9.7%)	10 (32.3%)	18 (58.1%)	4.484	0.6768	5	31
4…. increased my confidence to perform live horse castration.		1 (3.2%)	7 (22.6%)	8 (25.8%)	15 (48.4%)	4.194	0.9099	4	31
5. Simulation models should look and feel realistic in order to teach students technical skills.	1 (3.2%)	3 (9.7%)	7 (22.6%)	6 (19.4%)	14 (45.2%)	3.935	1.181	4	31
6. The equine castration model helped me identify my areas of strength and weakness for this skill.	1 (3.1%)		3 (9.7%)	13 (41.9%)	14 (45.2%)	4.258	0.8932	4	31
7. Performing castration on the model was easier than performing castration on the live horse.	2 (6.5%)	3 (9.7%)	12 (38.7%)	6 (19.4%)	8 (25.8%)	3.484	1.18	3	31
8. I feel confident to perform routine equine field castration under experienced supervision.				8 (25.8%)	23 (74.2%)	4.742	0.4448	5	31

**Figure 6:**
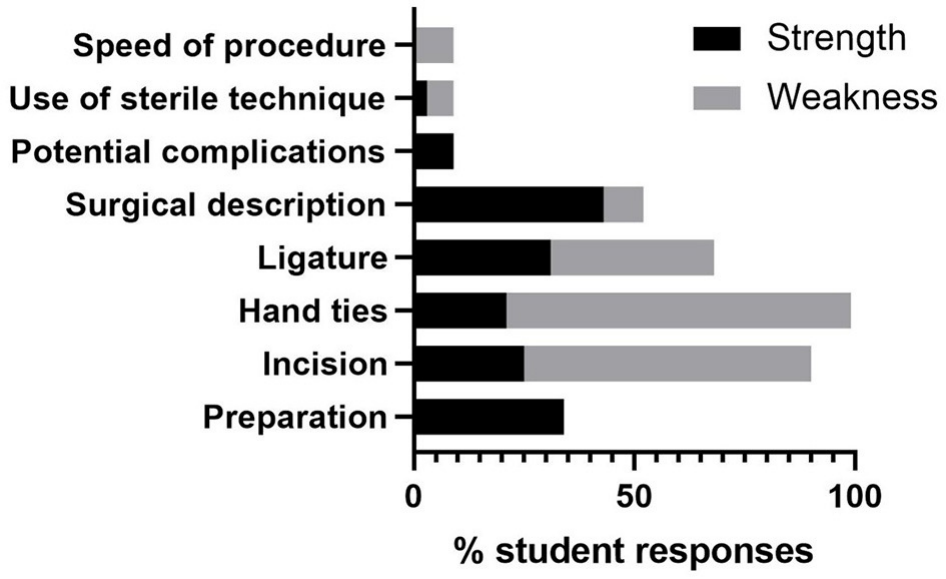
Percent of students identifying different categories of strengths and weaknesses for their equine castration model activity. Category codes were chosen to represent themes from student self-reflections on their own castration model video.

In the student self-reflections, the most commonly mentioned strengths were surgical description (14/32 students) and preparation (11/32 students), and the most commonly mentioned weaknesses were hand ties (25/32 students) and surgical incision (24/32 students) (Figure 6). These reflections are mostly consistent with rubric scores, as students commonly received “Exemplary” scores for verbal description of procedure and preparation and “Satisfactory” or “Development Required” for hand ties. However, there were also inconsistencies between student reflections and rubric scores, as raters were more likely to identify ligatures as a needed area for concentrated practice, rather than the incision (data not shown).

Seventeen of 32 student surveys provided written comments prior to live horse castration, while 21/32 students surveys provided written comments after live horse castration. There were three themes identified in the qualitative student feedback: model materials, recommendations for additional learning elements in the model, and value for student learning. For materials, student comments mentioned the silicone ball as being a good weight and feel to simulate a testicle, but that the tube sock meant to simulate the scrotum was hard to incise and the stocking meant to simulate the tunic “…*didn't hold suture well*.” Suggestions for additional learning elements included more spare parts for multiple practice opportunities, real emasculators, a way to simulate “…*stripping the fascia*…,” and a way to simulate common complications. Students made positive comments about the learning value of the model, stating that it was a safe way to practice, they gained confidence and skills, it improved their hand ties, and it was a good way to prepare for live horse castration. In terms of negative comments, there were students who stated that the requirement to make a video of themselves caused more anxiety than the model itself.

### Comparison of Student Confidence Survey

The results of student confidence surveys, with and without model experience, before and after live horse castration, are presented in Tables 4–7. Prior to live horse castration, there were no significant differences in confidence survey responses between students with vs. without model experience (data not shown). In the *post-live horse castration confidence survey*, students with model experience had significantly higher levels of confidence for “adequate preparation,” “confidence with general anesthesia,” and “confidence in hand ties,” compared to the group without model experience; but students with model experience had lower scores for “confidence during patient recovery” compared to the students with no model experience (Table 8). There were no significant differences in confidence survey results among students with different levels of castration experience (Tier I–III), either before or after live horse castration (data not shown). Because of small group size, this analysis was not performed for students who didn't use the model.

**Table 4:** Results of student confidence survey prior to live horse castration, with model experience.

**Survey item**	**Strongly disagree *n* (%)**	**Disagree *n* (%)**	**Neutral *n* (%)**	**Agree *n* (%)**	**Strongly agree *n* (%)**	**Mean**	***SD***	**Median**	***n***
I am technically competent to perform castration today.			1 (3.1%)	19 (59.4%)	12 (37.5%)	4.364	0.5488	4	33
I am mentally confident to perform castration today.			1 (3.1%)	22 (68.8%)	9 (28.1%)	4.273	0.5168	4	33
I am knowledgeable of the anatomical structures involved in equine castration.				19 (59.4%)	13 (40.6%)	4.424	0.5019	4	33
I am knowledgeable of the important history questions and post-op complications relevant to equine castration.			2 (6.3%)	21 (65.6%)	9 (28.1%)	4.242	0.5607	4	33

**Table 5:** Results of student confidence survey prior to live horse castration, no model experience.

**Survey item**	**Strongly disagree *n* (%)**	**Disagree *n* (%)**	**Neutral *n* (%)**	**Agree *n* (%)**	**Strongly agree *n* (%)**	**Mean**	***SD***	**Median**	***n***
I am technically competent to perform castration today.			1 (7.1%)	11 (78.6%)	2 (14.3%)	4.071	0.4746	4	14
I am mentally confident to perform castration today.			1 (7.1%)	7 (%)	6 (%)	4.357	0.6333	4	14
I am knowledgeable of the anatomical structures involved in equine castration.			1 (7.1%)	6 (%)	7 (%)	4.429	0.6462	4	14
I am knowledgeable of the important history questions and post-op complications relevant to equine castration.		1 (7.1%)	3 (31.4%)	6 (42.9%)	4 (28.6%)	3.929	0.9169	3	14

**Table 6:** Student confidence survey, post-castration, with model experience.

**Survey item**	**Strongly disagreed *n* (%)**	**Disagree *n* (%)**	**Neutral *n* (%)**	**Agree *n* (%)**	**Strongly agree *n* (%)**	**N/A *n* (%)**	**Mean**	***SD***	**Median**	***n***
I feel my preparation for today's procedure was adequate.			1 (3.3%)	7 (23.3%)	22 (73.3%)		4.7	0.535	5	30
Overall, I felt confident performing general anesthesia today.			3 (10%)	14 (46.7%)	12 (40%)	1 (3.3%)	4.345	0.6139	4	29
Overall, I felt confident performing equine castration today.				9 (30%)	21 (70%)		4.7	0.4661	5	30
I had anxiety while performing general anesthesia today.	3 (10%)	5 (16.7%)	7 (23.3%)	13 (43.3%)	1 (3.3%)	1 (3.3%)	3.103	1.113	3	29
I had anxiety while performing castration today.	3 (10%)	10 (33.3%)	10 (33.3%)	6 (20%)	1 (3.3%)		2.733	1.015	3	30
I would have known what to do today if my patient started waking up from anesthesia during surgery.			2 (6.7%)	24 (80%)	4 (13.3%)		4.067	0.4498	4	30
I felt confident while recovering my patient.	1 (3.3%)	1 (3.3%)	5 (16.7%)	12 (40%)	8 (26.7%)	3 (10%)	3.889	1.013	4	27
I was knowledgeable today on concepts of sterile technique.				8 (26.7%)	22 (73.3%)		4.733	0.4498	5	30
I was confident today in my knowledge of reproductive anatomy.			1 (3.3%)	10 (33.3%)	19 (63.3%)		4.6	0.5632	5	30
I was confident today in my hand-tie skills.			1 (3.3%)	16 (53.3%)	12 (40%)	1 (3.3%)	4.379	0.5615	4	29
I was confident today in my ligature placement and security.			3 (10%)	11 (36.7%)	15 (50%)	1 (3.3%)	4.414	0.6823	5	29
I would have known what to do today if my patient had bleeding from the surgical area.			1 (3.3%)	19 (63.3%)	10 (33.3%)		4.3	0.535	4	30
I am confident that I know how to give clients accurate instructions regarding discharge care, monitoring, and potential complications for equine castration.		1 (3.3%)	1 (3.3%)	11 (36.7%)	16 (53.3%)	1 (3.3%)	4.448	0.7361	5	29

**Table 7:** Student confidence survey, post-castration, no model experience.

**Survey item**	**Strongly disagree *n* (%)**	**Disagree *n* (%)**	**Neutral *n* (%)**	**Agree *n* (%)**	**Strongly agree *n* (%)**	**Mean**	***SD***	**Median**	***n***
I feel my preparation for today's procedure was adequate.			1 (7.1%)	9 (64.3%)	4 (28.6%)	4.214	0.5789	4	14
Overall, I felt confident performing general anesthesia today.		1 (7.7%)	2 (15.4%)	7 (53.8%)	3 (23.1%)	3.923	0.8623	4	13
Overall, I felt confident performing equine castration today.				6 (46.2%)	7 (53.8%)	4.538	0.5189	5	13
I had anxiety while performing general anesthesia today.		4 (30.8%)	6 (46.2%)	1 (7.7%)	2 (15.3%)	3.077	1.038	3	13
I had anxiety while performing castration today.	3 (23.1%)	7 (53.8%)	1 (7.7%)	2 (15.3%)		2.154	0.9871	2	13
I would have known what to do today if my patient started waking up from anesthesia during surgery.			2 (15.3%)	6 (46.2%)	5 (38.5%)	4.231	0.725	4	13
I felt confident while recovering my patient.				6 (46.2%)	7 (53.8%)	4.538	0.5189	5	13
I was knowledgeable today on concepts of sterile technique.			1 (8.33%)	4 (33.33%)	7 (58.33%)	4.5	0.6742	5	12
I was confident today in my knowledge of reproductive anatomy.				5 (41.7%)	7 (58.3%)	4.583	0.5149	5	12
I was confident today in my hand-tie skills.		4 (33.33%)	3 (25%)	5 (41.66%)		3.083	0.9003	3	12
I was confident today in my ligature placement and security.				5 (41.7%)	7 (58.3%)	4.583	0.5149	5	12
I would have known what to do today if my patient had bleeding from the surgical area.			3 (25%)	3 (25%)	6 (50%)	4.25	0.866	4.5	12
I am confident that I know how to give clients accurate instructions regarding discharge care, monitoring, and potential complications for equine castration.			2 (16.7%)	3 (25%)	7 (58.3%)	4.417	0.793	5	12

**Table 8:** Significant differences in student confidence survey responses in model vs. no model, post-live horse castration.

	**With model experience**	**No model experience**	***P-*value**
	**Median (Q1–Q3)**	**Median (Q1–Q3)**	
I feel my preparation for today's procedure was adequate.	5 (4–5)	4 (4–5)	0.007
I was confident today in my hand-tie skills.	4 (4–5)	3 (2–4)	<0.0001
I felt confident while recovering my patient.	4 (3–5)	5 (4–5)	0.0442

### Expert Feedback on Model

**Figure 7:**
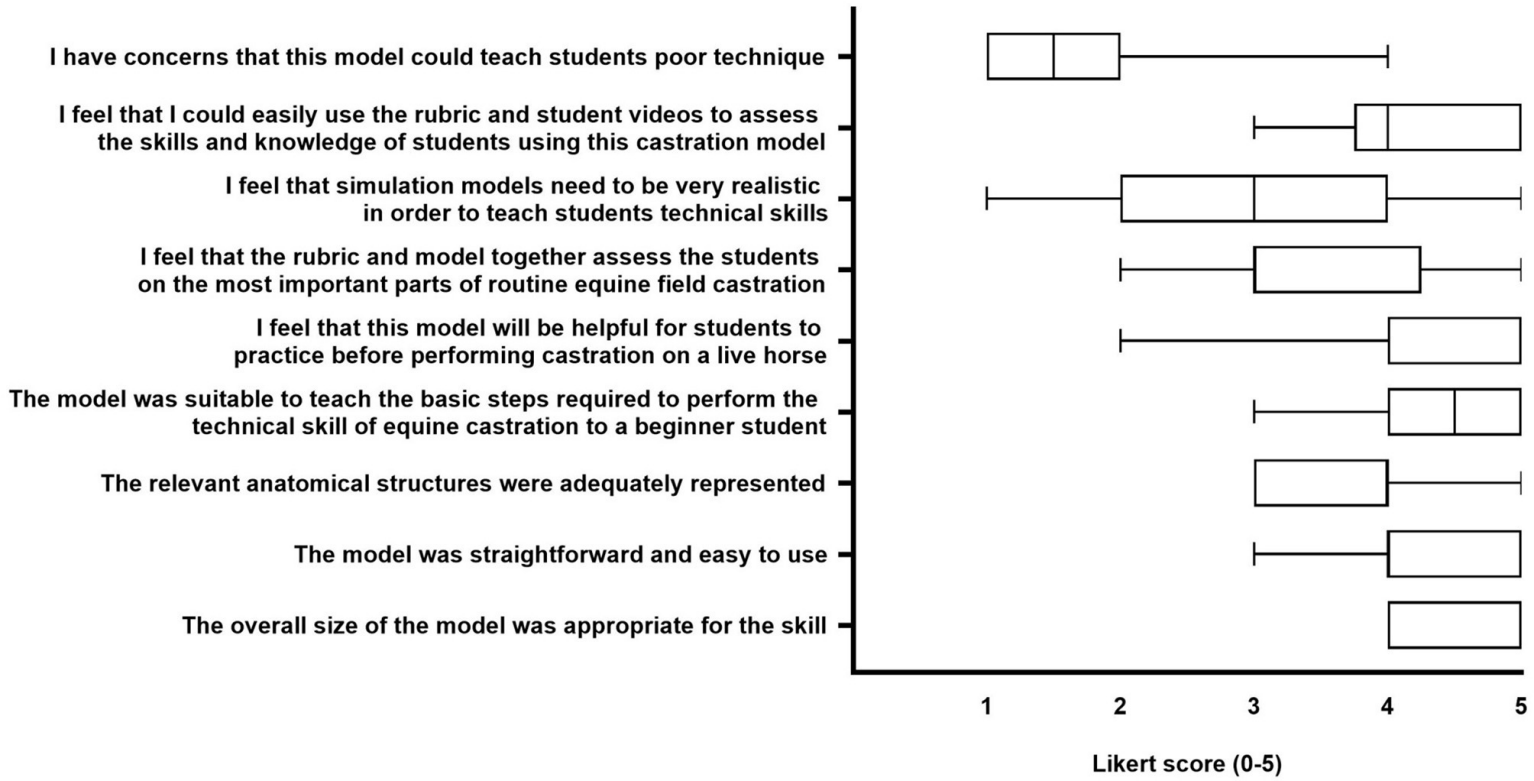
Survey results of expert veterinary evaluation of the low-fidelity equine castration model. Data are presented as median and min-max.

We received expert feedback on the equine castration model from 10 ACVS boarded academic faculty, 1 large animal surgery resident, and 1 equine intern. None of the experts providing feedback were involved in the design or evaluation of the model or rubric. Survey responses indicated that experts felt the model would have educational and assessment value for veterinary students (Figure 7). Of the four written comments, one expert recommended adding emasculators to the model experience, and another recommended finding a way to prevent the plastic container from sliding on the tabletop.

### Rubric Evaluation

There were a total of 22 scores from Reviewer A, 24 scores from Reviewer B, and 22 scores from Reviewer C. Reviewer A and B had 14 paired scores, Reviewer A and C had 11 paired scores, Reviewer B and C had 10 paired scores. There were 8 students that received a score from all three reviewers.

There were no significant differences in the median scores of Reviewers A, B, and C (Kruskal-Wallis, *p* = 0.5737). For the 8 cases that were rated by all 3 reviewers, the agreement was moderate with an ICC of 0.447 (*p* = 0.0238). Looking at it pairwise (where there is a larger sample number) the ICCs were higher. For Reviewers A and B, we saw an ICC value of 0.622 (*p* = 0.00668, *n* = 14), for B and C a value of 0.563 (*p* = 0.0282, *n* = 11), and for A and C a value of 0.634 (*p* = 0.00178, *n* = 18).

The pairwise values being uniformly higher than the three-way says that we would expect the true ICC to be higher than what we see in the three-way and that more samples would support that conclusion. The percent agreement in pairwise comparisons of reviewers are shown in Table 9. While percentage of perfect score agreement was 50% or less, the within 1 agreement was >70% for all pairwise comparisons.

**Table 9:** Rubric performance.

	**Mean (*SD*)**	**Median (Q1–Q3)**	**100% agreement**		**Within 1 agreement**		**ICC**, ***p*****-value**	
			**Reviewer B**	**Reviewer C**	**Reviewer B**	**Reviewer C**	**Reviewer B**	**Reviewer C**
Reviewer A	13.32 (1.323)	13.5 (12.75–14)	21.4%	0.09%	92.9%	81.8%	0.622, *p* = 0.00668	0.634, *p* = 0.00178
Reviewer B	13.6 (1.113)	14 (13–14.38)	–	33.3%	–	72.2%	–	0.563, *p* = 0.0282
Reviewer C	13.14 (1.612)	14 (12–14)	–	–	–	–	–	–

## Discussion

In this paper, we describe the development, implementation, and student and expert assessment of a low-fidelity equine castration simulator and associated student learning activities. Knowing that authentic clinical experience can inform student perspectives on previous educational experiences (18), we surveyed student attitudes toward the model both before and after live horse castration. Prior to live horse castration, students with the least experience (Tier I) had more favorable attitudes toward the model for increasing knowledge of “the surgical procedure” and “key anatomical structures,” compared to students with the most experience (Tier III) (Figure 4). This difference was not observed in the survey following live horse castration (data not shown). It makes sense to us that students with less practical experience would benefit most from simulator experience; however, this finding is in contrast to previous reports that medical students with limited experience may not be able to appreciate what they actually learned from a simulation (18). After live horse castration, total student responses were significantly more favorable toward the model for “increasing knowledge of key anatomical structures” and being “helpful for learning the surgical technique of routine equine castration” (Figure 5). This finding is consistent with Bewley and O'Neil, suggesting that authentic clinical experience helps all students see more value in model training (18).

In order to have long term success in veterinary medicine, practitioners must be lifelong and experiential learners. The process of experiential learning is described by David Kolb's four “learning modes,” in which learners simultaneously experience the outer world, reflect on their experiences, use these reflections to build their inner world knowledge and memories, and decide how to act/be/know/do in the outer world (19). We included reflection as a component of the equine castration model in order to encourage students to conduct their own self-assessment of their performance. However, the qualitative nature of their reflection makes it difficult to ascertain whether students feel they exceeded, met, or fell below expectations. Moving forward we plan to better emulate Kolb's Experiential Learning model by asking students to not only reflect on the strengths and weaknesses of their performance, but to rate themselves regarding performance expectations, and submit a plan for how they will address skills that fell below expectations, prior to live horse castration.

One interesting finding of our study was that most students did not report high levels of anxiety regarding live horse castration, whether they had model experience or not. In fact, although not significantly different, students without model experience trended toward having less anxiety than students with model experience. It is important to note that the students in our study were enrolled in an equine elective that is usually only taken by students with prior equine handling experience and equine career aspirations, which could have contributed to their lack of anxiety. However, based on our previous experience teaching live horse castration in this course, we had hypothesized that students would have moderate to high levels of anxiety toward live horse castration. There are two different models of learning and skill development that could also inform the lack of anxiety reported by our students (20). The Hersey-Blanchard Situational Leadership model proposes four levels of learner development from avid beginner, disillusioned beginner, reluctant learner/cautious contributor, to expert/self-reliant achiever (20). These learner categories are paralleled in the four Stages of Competence Model: unconscious incompetence, conscious incompetence, conscious competence, or unconscious competence (21). In both of these models, learners begin with limited skills. According to Burch, novice learners are unaware of their knowledge or skills gaps (unconscious incompetence) (21). In the Hersey-Blanchard model, novice learners are enthusiastic and self-motivated (avid beginner). As learners progress in competence, they remain unskilled, but they become aware of their deficits (conscious incompetence). In the Hersey-Blanchard model, these second stage learners may have lower motivational levels due to difficulties they have encountered with learning (disillusioned beginner). We propose that students in our no model group, which included 1st through 3rd years, were unconsciously incompetent. Because of minimal practical experience or training, they had little anxiety about performing the procedure. Any anxiety they did have could have been overshadowed by the excitement toward performing a new skill. Interestingly, compared to students without model experience, students with model experience, who had completed higher-level medicine and surgery courses, felt significantly less confident while monitoring anesthetic recovery of a live horse. This difference could illustrate increased learner self-awareness and progression to conscious incompetence.

Based on our survey results, students *with* vs. *without* model experience had significantly higher confidence in “adequate preparation,” “confidence with general anesthesia,” and “confidence in hand ties.” The increased confidence in general anesthesia is likely explained by the fact that third year veterinary students have had significantly more instruction on this topic than first- or second-year students. The increased confidence in adequate preparation is likely due to instructional opportunities, as well as model experience. The increased confidence in hand ties may or may not be related to model experience. Third year students in our study may have had other extra-curricular opportunities to practice hand ties. However, equine faculty recognize the need for increased instruction and practice opportunities for hand ties in the DVM curriculum, since first year simulation labs and second year surgery courses tend to focus on training students with surgical instruments. This equine castration model provides an important “touchpoint” within our curriculum to emphasize the importance and utility of hand ties for equine-oriented students.

Development of novel educational tools and methods is an iterative process. Based on student and expert feedback, we have made, and plan to make, changes to the described equine castration model. First, a new instructional video has been developed in order to show students how to assemble the model correctly. Our intent with this resource is to address student concerns regarding difficulty with ligature placement due to cord length. Additionally, to better simulate the “springy-ness” of the cord, the looped Kling was replaced with a latex tube. Theoretically, this will allow students to better appreciate the quality of their ligatures. Finally, the instructional video suggests that student use duct tape to secure the plastic container to the table-top to keep it from sliding.

Both students and experts suggested adding emasculators to the equine castration model simulator. While our students were instructed to describe emasculator placement as part of their surgical description, our model does not currently include an item meant to model this equipment. Interestingly, some students did use common household items (i.e., pliers) to simulate emasculators. Emasculators are routinely used for equine field castration and learners often have no previous exposure to this piece of surgical equipment. Indeed, students are often surprised by the amount of strength and force required to close emasculators and it would be ideal for them to have this experience prior to live horse castration. One option to add a low-fidelity “emasculator” to this model would be to use locking or self-locking clamps or pliers with an added “wingnut” and designated “cutting blade” to allow students to demonstrate proper orientation of the emasculator. The addition of a low-fidelity emasculator would encourage students to “mentally rehearse” the correct placement and timing of this important step of equine castration. One downside to this addition would be increased cost. Additionally, it would not help prepare students for the physicality of emasculator placement, which is often their biggest struggle. Moving forward, we do plan to offer a separate simulation experience in which students can, under supervision, practice using emasculators on tissue.

One limitation of this study is that our data does not provide evidence for the overall validity of this model (22). For our initial description and investigation of this model, we focused on content-related validity by surveying student attitudes (face validity) and expert feedback (construct validity). Further investigation relating learner performance on this model to other similar measures (concurrent validity) and subsequent competence in live horse castration (predictive validity) is needed to determine whether the model has criterion-related validity (23). However, because there is no previous description of an equine castration model in the veterinary education literature, we felt the report of this preliminary investigation would be of potential value to veterinary educators and students alike.

Another limitation of our study is the small number of student participants, which precluded our ability to perform more formal statistical evaluation of the rubric with measures such as Cronbach's alpha. Rubric performance could also have been affected by the video and audio of the assignment, which precluded our ability to perform blinded evaluation, thereby increasing the risk of rater bias (24). This is one potential explanation for our acceptable, but not exceptional, interrater reliability. Other potential reasons for our interrater reliability include lack of standardized training for raters and the multiple levels within the rubric design (24). Other measures of reliability, such as intra-rater reliability, were not addressed in this study. Although our interrater reliability could be improved, our purpose in creating this activity was not to design a highly standardized, high-stakes, summative assessment in which rater scores should have high reliability and validity standards (24). Instead, our goal was to create an affordable simulator and associated formative assessment that allowed students a chance to: (1) practice a cognitive, step-wise approach to equine field castration, (2) recognize their own strengths and weaknesses when performing a clinical procedure, and (3) receive timely and specific expert feedback on this skill. Our goal with this design method was to decrease the extraneous cognitive load students often experience during direct instructor observation. While this seemed to work for most students, a few students reported that making the video was the most stressful part of the exercise. However, the video was an integral aspect of the overall learning activity, because we asked students to explain the steps of castration aloud. Thinking aloud while performing clinical exams or procedures is one way that instructors and learners can make their thinking obvious (25). When instructors think aloud, students hear a model for how an expert processes and reasons with information in real time. When students think aloud, instructors have a window into a learner's understanding, awareness, and ability to reason through more complex clinical tasks and problems. Thinking aloud also reinforces a learner's own understanding. The think aloud aspect of this simulator activity, along with the learner self-reflection, are two ways we designed this assessment to be not only *of* student learning, but *for* student learning (26). In order to further develop the formative nature of this activity, we plan to simplify the rubric skills assessment to “competent” or “not yet competent.” We will then have a follow up activity asking students to submit a written plan explaining how, when, and how often they will practice their “not yet competent” skills prior to live horse castration.

This manuscript describes development of a novel, low-fidelity equine castration model, and associated learning activities for third year veterinary students, and the results of student and expert formative feedback. Overall, our findings suggest that both students and veterinary educators feel that this low-fidelity model has educational value and content validity. Based on the feedback received, we have already implemented changes to the model and plan to implement changes to the rubric, including standardization of instructor training and simplification of rubric levels. Future research will investigate the criterion validity of this model, including whether number of models completed impacts student competence score during live-horse castration.

## Data Availability Statement

The raw data supporting the conclusions of this article will be made available by the authors.

## Ethics Statement

The studies involving human participants were reviewed and approved by North Carolina State University Institutional Review Board. The patients/participants provided their written informed consent to participate in this study.

## Author Contributions

MS, MB, KF, and CF contributed equally in the project planning and study design. CF and KF designed the castration model. KF produced and assembled the models. CF, MB, and MS designed learning activities to support the model and evaluated castration videos. MS designed the surveys, submitted the IRB, and analyzed the survey data. JR analyzed and helped to interpret the data. All authors contributed to manuscript preparation and revision.

## Funding

Publication fees supported in part by NC State CVM Research Funds and Department of Clinical Science.

## Conflict of Interest

The authors declare that the research was conducted in the absence of any commercial or financial relationships that could be construed as a potential conflict of interest.

## Publisher's Note

All claims expressed in this article are solely those of the authors and do not necessarily represent those of their affiliated organizations, or those of the publisher, the editors and the reviewers. Any product that may be evaluated in this article, or claim that may be made by its manufacturer, is not guaranteed or endorsed by the publisher.
