# Supplementary material for: Development and Formative Evaluation of a Low-Fidelity Equine Castration Model for Veterinary Education
Source: Front Vet Sci. 2021 Sep 14;8:689243. doi: 10.3389/fvets.2021.689243 (PMC8476848; doi:10.3389/fvets.2021.689243)
Supplement: Supplementary file 1 [file Table_1.docx]

**CASTRATION PRE-LAB EXERCISE**

These exercises make up 40% of your grade in this course. Please take them seriously, and contact me with any questions or concerns EARLY in the semester. Please do them independently.

1. Reading assignment: Read and understand the castration procedure and related material within the uploaded chapters.
   1. Learning objectives for the reading: Understand why castration is needed and how it is used in the delivery of care, details of different castration options and when those would be useful. Be able to list common castration complications, symptoms for each, preop/intraop methods for preventing them, and how to treat each.
2. Complete and pass quiz based on the above learning objectives.
3. Develop a written step-by-step description that includes a complete supply list, how to prepare/evaluate a colt for castration, induction and monitoring of anesthesia, and how to perform a field recovery.
4. Develop a written step-by-step detail of the surgical procedure from beginning to end, including how to check the emasculators for proper assembly.
5. Develop and submit an anesthetic protocol, based on the scenario given. Use your Anesthesia notes as a guide.
6. Read and understand the Castration model rubric and the Live horse castration rubric, and watch the castration model videos (audio first then no audio), and watch the hand tie videos prior to beginning practice with the model.
7. Practice with the model several times, until you are comfortable with each of the components that you will be evaluated on (those can be found in the rubric). Then, make a video of yourself performing the procedure on the castration model, while talking through each of the steps. **Include a self-reflection component at the end of the video in which you verbally give **two** strengths and **two** areas in which you believe you could benefit from concentrated practice, based on the video performance** . Phone videos are fine, or you can borrow a Flipcamera from the library to video. Please contact xxxx if you have trouble uploading the video.
8. Watch the live horse castration video. Answer the Moodle questions that follow. Identify the castration method, and give a critique of the performance. Give two strengths and two areas for concentrated practice for this surgeon. Identify at least two technical errors.
9. Submit all files in Moodle, by xxxx, in order to participate in the castration labs.
